# Observations of Delayed Changes in Respiratory Function among Allergy Clinic Patients Exposed to Wildfire Smoke

**DOI:** 10.3390/ijerph19031241

**Published:** 2022-01-22

**Authors:** James Blando, Michael Allen, Hadiza Galadima, Timothy Tolson, Muge Akpinar-Elci, Mariana Szklo-Coxe

**Affiliations:** 1 School of Community and Environmental Health, Old Dominion University, Norfolk, VA 23529, USA; hgaladim@odu.edu (H.G.); mszklo@odu.edu (M.S.-C.); 2 Geography Program, Old Dominion University, Norfolk, VA 23529, USA; mallen@odu.edu; 3 Albemarle Allergy & Asthma PC, Elizabeth City, NC 27909, USA; tatmd0316@gmail.com; 4 School of Public Health, University of Nevada, Reno, NV 89557, USA; makpinar@unr.edu

**Keywords:** wildfire, peak flow, sensitization, smoke, respiratory, allergy, delayed

## Abstract

Wildfires have increased in frequency and magnitude and pose a significant public health challenge. The principal objective of this study was to assess the impact of wildfire smoke on respiratory peak flow performance of patients exposed to two different wildfire events. This longitudinal study utilized an observational approach and a cohort study design with a patient-level clinical dataset from a local outpatient allergy clinic (*n* = 842). Meteorological data from a local weather station served as a proxy for smoke exposure because air quality measurements were not available. This study found that there were decreases in respiratory peak flow among allergy clinic patients one year after each wildfire event. For every one percent increase in wind blowing from the fire towards the community, there was, on average, a 2.21 L per minute decrease in respiratory peak flow. This study observed an effect on respiratory peak flow performance among patients at a local allergy clinic one year after suspected exposure to wildfire smoke. There are likely multiple reasons for the observation of this relationship, including the possibility that wildfire smoke may enhance allergic sensitization to other allergens or that wildfire smoke itself may elicit a delayed immune response.

## 1. Introduction

Climate change has impacted a wide range of environmental phenomena, including wildfires. Many studies have found that the increasing drought conditions, changes in fire season length, alterations in the timing of snowmelt, changes in precipitation, and heat experienced by many places around the globe contribute to more frequent and larger wildfires [1,2,3,4,5]. The public health impact on communities exposed to wildfire smoke has been evaluated and suggests potentially significant effects [6,7,8,9,10,11,12,13,14,15,16]. Arriagada et al. [9], in their meta-analysis, found that asthma admissions were associated with exposure to wildfire smoke and that females were more susceptible than males. Delfino et al. [8] similarly found elevated rates for several respiratory conditions and also found that in some subgroups, there appeared to be an elevated risk in the two-week period after the wildfire. Orr et al. [17] found a decrease in pulmonary function among residents exposed to wildfire smoke 1 to 2 years after exposure.

In 2008 and 2011, residents of northeastern North Carolina were impacted by wildfire smoke as a result of two significant peat bog fires in the Great Dismal Swamp National Wildlife Refuge (https://www.fws.gov/refuge/great_dismal_swamp/, accessed on 31 December 2021). There were also wildfires that occurred in other areas (Pains Bay Fire and Pender County Fire; see [18]) before the Dismal swamp fire of 2011 and one that occurred concurrently with the Dismal Swamp fire of 2008 in the Pocosin Lakes National Wildlife Refuge (see [6]). To our knowledge, there were no other major wildfires in this area from 2007 through 2012 based on internet searches and data from the National Interagency Fire Center (https://www.nifc.gov/index.html, accessed on 31 December 2021).

We endeavored to review the medical records at a local outpatient allergy clinic in order to examine potential effects on community members, with a specific focus on the Dismal Swamp fires. 

## 2. Materials and Methods

For this study, access to medical records was crucial, and as such, the identification of a clinic in an area impacted by the Great Dismal Swamp wildfires had to be identified. The participating clinic was in a small town that can be characterized as an urban cluster within a county of less than 40,000 residents. The clinic was located southeast of the fire area and roughly 25 miles from the center and approximately 10 miles from the southern border of the Great Dismal Swamp National Wildlife refuge. This study was approved by the University Institutional Review Board (IRB) under protocol #1207671-4.

This study utilized an observational approach for the initial exploratory analyses and a cohort study design for the final analyses. Initially, a descriptive assessment of the data was completed by looking at the relative frequency of the ICD-9 codes assigned at the clinic during the pre-fire, during-fire, and post-fire periods to determine which ICD codes occurred more frequently in each time period. Then, patient-level data for common ICD codes from the participating clinic’s medical charts were abstracted for the years 2007 through 2012 and included patients seen at the clinic during this five-year period (total 10,024 clinic visits), their demographic information, age, height, weight, date of clinic visit, primary and secondary ICD-9 codes, peak flow measures, predicted peak flow, and their personal best peak flow measure recorded at the clinic as a baseline. Each patient had their BMI in kg/m^2^ at the time of their clinic visit computed as per standard methodology [19]. This longitudinal data followed the patients over the five-year study timeframe and therefore, many patients had repeated peak flow measures recorded over time. Data were analyzed separately for each fire event, where the first fire (Fire #1) occurred from 9 June through 7 October 2008 and the second fire (Fire #2) occurred from 4 August through 11 October 2011. The datasets for each fire event were grouped for analysis into time periods that included a pre-fire period, during-fire period, and post-fire period. The pre-fire period was the same calendar days as the fire but one year prior to the fire event. For example, the pre-fire period for Fire #1 was therefore 9 June through 7 October 2007. The during-fire timeframe was when the wildfire was actually burning, and the post-fire time period was the same calendar days as the fire event but one year after the fire had occurred. For example, the post-fire period for Fire #1 was therefore 9 June through 7 October 2009. The rationale for the selection of these time periods was that they would be the most comparable time periods because throughout the year, other airborne agents, such as allergies, viral infections (e.g., flu season), and air pollutants (e.g., pollen and molds, ozone, etc.), vary seasonally and impact clinic visits and the respiratory performance of patients. Having the datasets match on calendar days helps to roughly adjust for this seasonal variation and therefore makes the patient data more comparable because they are compared during the same season with the same seasonal influences.

Several clinical metrics were also computed from the raw data. The peak flow measures recorded at the clinic were the best of three attempts by the patient during their clinic visit. This involved the patient blowing into a peak flow meter to measure the maximum flow rate produced in liters per minute during exhalation. This technique is a standard clinical measure indicative of airway obstruction, such as that which occurs during an asthma exacerbation, but it may underestimate the degree of obstruction [20]. In addition to the patient’s actual peak flow measure during their clinic visit, a derived value was also computed that compared the clinically measured peak flow of the patient to that which would have been expected and to the patient’s baseline personal best. This value was computed and represented by a variable referred to as “delta_PF”, which was calculated by subtracting the peak flow expected for the patient from the actual peak flow measured at the clinic visit. Another derived variable referred to as “delta_Best” was the actual peak flow measured during the clinic visit minus their personal baseline best peak flow value. If the patient was performing below that which would have been expected or below their personal best, this delta value would be negative, and the more negative the value, the greater the deficit in the patient’s respiratory performance. These computed peak flow values were necessary to allow for aggregate analysis of patient performance across the wide range of demographics of the patients in the dataset, which included patients of all ages, both sexes, and diverse ethnic and racial groups.

**Figure 1:**
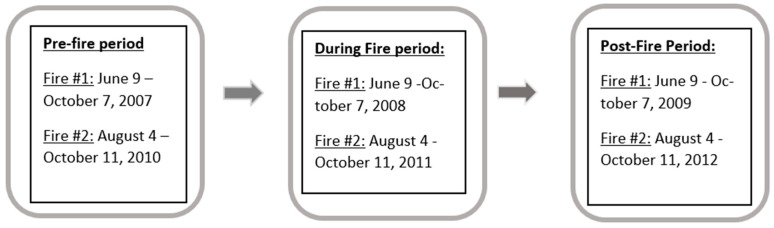
Patient dataset time periods of interest (January 2007–December 2012).

Statistically, the patient-level data were analyzed longitudinally using three different time periods, including the pre-fire, during-fire, and post-fire time periods (Figure 1). First, an observational approach where data were descriptively assessed for all patients and each demographic group, using SAS v9 Proc Means, was used. Second, a repeated measures analysis of variance (ANOVA) was run across the three time periods for each fire event to assess differences in the mean delta_PF between groups and assess a pairwise comparison of the different time periods using a Tukey–Kramer adjustment for each subject in the longitudinal data. This was carried out using Proc Mixed in SAS v9. Third, the primary final analyses used a cohort study design, where each fire had its own cohort of patients who had peak flow measures in all time periods of interest, which included the pre-fire, during-fire, and post-fire time periods (Figure 1). In other words, we had two cohorts that were analyzed separately, one for Fire #1 and another for Fire #2. Each cohort was analyzed separately using linear regressions that were run to assess the post-fire periods for a delayed respiratory effect. Clinical peak flow metrics for the post-fire period used delta_best as an outcome. The primary predictors included in the model were meteorological measurements of wind direction from the fire, age, BMI, race, and sex. The meteorological measurements served as a proxy for exposure to smoke and were taken from the local coast guard station located in the same city as the clinic, and included a measure of the percentage of time the town was downwind of the active wildfire for the three days prior to a patient’s clinic visit during the fire. Wind direction and wind speed were the best proxies for exposure to smoke available because satellite data were either not available for the time periods of interest or not feasible to use due to a lack of resolution and an inability to distinguish between smoke particles from the wildfire and other common ambient particles in the air, such as soil dust, car exhaust, or water droplets. Only patients that had clinical measurements during all three periods of pre-fire, during fire, and post-fire were included in this analysis. Therefore, the clinical peak flow measurements were in the post-fire period, but their exposure proxy to the wildfire smoke was the meteorology they were exposed to during the actual fire event. Unadjusted as well as adjusted models were run.

## 3. Results

The clinic’s patient population was primarily derived from the local areas surrounding the clinic. Specific patient demographics are provided in Table 1. The assessment of ICD codes in the patient medical charts suggested that the most frequent respiratory system diagnoses at this local clinic during the fire periods were essentially allergic rhinitis (36% for Fire #1 and 30% for Fire #2) and asthma (8% adult Fire #1 and 15% children Fire #1, 10% adult Fire #2 and 15% children Fire #2). Many patients had multiple diagnoses: for patients with allergic rhinitis, approximately 80% during Fire #1 and 90% during Fire #2 also had a diagnosis of asthma. Therefore, all analyses presented below focused only on patients with an allergic rhinitis and/or asthma diagnosis.

Descriptive statistics showed that the average change in peak flow measures across the pre-fire, during-fire, and post-fire periods was greatest in the post-fire period. Table 2 shows the average delta_PF (clinic measured peak flow minus their predicted peak flow) data across all periods for Fire #1 for all patients with a diagnosis of allergic rhinitis and/or asthma. Table 2 descriptively demonstrates that the greatest negative change in peak flow from predicted peak flow was observed in the post-fire period (−60.43 L/min) and the greatest decrease was among women (−75.65 L/min) and among black patients (−73.36 L/min). The same patterns occurred when assessing data from Fire #2, however, the post-fire period in Fire #2 had a slightly smaller negative delta_PF (−55.33 L/min) compared to Fire #1.

The repeated measures ANOVA with pairwise comparisons in Table 3 also suggested that delta_PF values were the most negative in the post-fire period relative to the during-fire and pre-fire periods. Data gathered from both Fire #1 and Fire #2 on patients who had clinic measured peak flow values across all three time periods (pre-, during, and post-fire) indicated that pairwise comparisons of the difference between the clinically measured peak flow vs. the predicted peak flow (delta_PF) were significantly different in the post-fire period when compared to the pre-fire or during-fire periods for Fire #1 and marginally significant for the during-fire vs. post-fire periods for Fire #2 (Table 3). The same pattern was observed for the clinically measured peak flow vs. their personal best (delta_best) outcome variable.

Third, an analysis using adjusted multiple linear regression with the post-fire clinical data and meteorological parameters during the wildfire events was conducted. In the multivariate models, we included five variables, which included the meteorological parameter of percentage of time the town was downwind of the fire (a proxy for exposure to wildfire smoke during the fire period) and the post-fire period demographics of age, BMI, sex, and race as covariates. In this case, analyses were conducted on the delta_best variable as the outcome. The model can be represented by the equation:

Delta_best = β_0_ + β_1_X_1_ + β_2_X_2_ + β_3_X_3_ + β_4_X_4_ + β_5_X_5_ + ε (1)

where: 

β_0_ = intercept

β_i_ = slope of variable X_i_, where i = 1, 2, 3, 4, or 5

X_1_ = percentage of time town was downwind of the fire

X_2_ = age

X_3_ = body mass index of patient

X_4_ = sex of patient

X_5_ = race of patient

ε = random error

Statistically significant associations with negative β coefficients were found between the delta_best outcome variable and the percentage of time the wind was blowing from the fire towards the affected area (i.e., town was downwind of fire) during the wildfires for the three-day average time period. More specifically, the results show a statistically significant decrease of 2.21 L per minute in delta_best for each percentage point increase in wind blowing from the fire towards the town (*p* = 0.032) while adjusting for all other variables in the model. The most significant decrease in delta_best was noted in association with a strong wind (Beaufort wind scale > 6) blowing from the fire towards the town (β = −13.90, *p* = 0.0060). The data for the adjusted multivariate model are summarized in Table 4.

## 4. Discussion

This study found that the patients visiting an allergy clinic located in an area impacted by wildfire smoke had a significant decline in the peak flow performance one year after the wildfires occurred. The descriptive metrics used suggested that women and African-American people appeared to have the most negative average delta_PF (difference between clinic visit value vs. predicted) compared to other demographic groups. For example, the descriptive values observed an average of 76 L per minute lower clinic measured peak flow than that which would be predicted among the women in the post-fire period (Table 2). If this were, for example, a 30-year-old female that is 5′5” in height with a 448 L per minute peak flow [21], it would represent roughly a 17% drop in performance. Descriptive statistics showed that Fire #1 had more significant decreases in delta_PF compared to Fire #2, and this could be because Fire #1 lasted longer than Fire #2 and hence there was more exposure. It could also be a result of the smaller sample size of patients in Fire #2, which had an impact on the computed average delta_PF values. It was also noted that the post-fire period had more negative delta_PF values than the during-fire and pre-fire periods. This could have occurred for several reasons, including the smaller sample size of the post-fire group resulting in averages more subject to the influence of outliers. It is also possible that a potential biological mechanism for this observation was that patients were sensitized to allergens in the smoke released from the wildfire and this sensitization resulted in a delayed response, hence the observation of the most negative delta_PF values in the post-fire period. However, as Table 2 is simply descriptive in nature, further statistical testing was required. 

The initial data and existing literature suggested that looking at delayed effects might be worthwhile. As a result, we conducted a repeated measures analysis longitudinally comparing the different time periods among the data for Fire #1 and different time periods among the data for Fire #2. Table 3 summarizes the results of this repeated measures ANOVA test with pairwise comparisons among the patients in the study and showed that the pre-fire and during-fire periods were not significantly different from each other, but the post-fire period values were significantly different from the pre- and during-fire time periods for Fire #1 (Table 3). Fire #1 had more than twice the number of patients compared to Fire #2 and therefore had higher statistical power to detect differences. The values for Fire #2 comparing the during-fire and post-fire periods also approached a statistically significant difference. This indicated that the delta_PF values seen in the post-fire period were significantly more negative than the other periods. This analysis indicated that the post-fire period had a detectable statistically significant deficit in delta_PF and that the post-fire period, which was one-year after the wildfire event, could be impacted. This is conceivable as there are a number of reports in the literature that demonstrated that it may be possible for ambient air pollution, and wildfire smoke specifically, to act as an adjuvant to a delayed allergic sensitization of the respiratory system [22,23,24,25,26,27,28,29,30,31]. Therefore, the allergy patients attending this clinic may have become more susceptible and experienced more significant changes in peak flow associated with their allergic rhinitis and asthma as a result of their wildfire smoke exposure, and this was detected one year after the wildfire event. 

The data in Table 4 showed that during Fire #1, there were statistically significant findings between several meteorological parameters and post-fire delta_best clinic measures, whereas Fire #2 did not have significant findings. This again could be due to the larger sample size for Fire #1. However, both Fire #1 and Fire #2 consistently had negative β values. In fact, Fire #1 had the largest statistically significant negative β values for a light (Beaufort 2–3 = 1.5–5.5 m/s) and strong wind (Beaufort >6 = >10.7 m/s) coming out of the northwest (winds blowing from the Great Dismal Swamp fire towards the town). The values in Table 4 show some β values that are positive, but the positive β values were not statistically significant, rather only some of the negative β values were statistically significant. The meteorology of a coastal site is complex and the observations in Table 4 were not meant to serve as an assessment of this complexity but rather as simple observations. Overall, the data from the regression models for a wind blowing from the fire towards the town had a *p*-value of 0.03 and a β value of −2.21. The β value was interpreted to mean that for every increase in percentage of time the wind was coming from the fire towards the town, there would be a decrease in delta_best of 2.21. In other words, each percentage increase in wind from the fire resulted in an additional decline of 2.21 L per minute in peak flow among the patients at the clinic one year after the fire ended. Using our example of a 30-year-old women that is 5′5” in height, this would be a 0.5% decline in peak flow one year after the fire for each percentage increase in wind coming from the wildfire towards the town. Interestingly, the data also suggested that the decreased patient peak flow remained after adjustment of the models for the covariates of age, race, BMI, and sex. The variable for percentage of time the wind was blowing from the fire towards the town remained significant after model adjustment, which indicates that the relationship is robust and that the results cannot be explained simply by the covariates of age, race, BMI, and sex. The relationship between wind direction as a proxy for exposure to wildfire smoke and its association with decreases in respiratory peak flow one year after exposure among patients at the allergy clinic appears to be robust. 

This study had several strengths and limitations. This study evaluated patients that visited a local allergy outpatient clinic for care and therefore were much less acute in their illness than patients admitted to the hospital or those going to the emergency department. Patients going to the hospital represent the sickest patients and represent a smaller proportion of the population than those going to local clinics for care. Therefore, this study provides data representative of the larger background population, albeit those with pre-existing allergies. This study also provides insights using clinical measures of respiratory performance and therefore may represent the potential impact of wildfire smoke on the respiratory system. This study also utilized repeated measures on the patients evaluated and therefore provided a longitudinal assessment of the associations investigated. As noted previously, there were also three other wildfires that occurred during the years of the Dismal Swamp wildfires. However, the Pains Bay and Pender County fires of 2011 occurred before the Dismal Swamp fires and the timeframe of those fires was not included in the analysis. The Pocosin Lakes National Wildlife Refuge fire occurred concurrently with the 2008 Dismal Swamp fire, but this fire was located southeast of the clinic location, meaning that the wind direction analysis would not be confounded because when the wind was coming from the Dismal Swamp, the smoke from the Pocosin fire was blowing away from the study area. This study was limited in that actual air pollution measures in the North Carolina community examined were not available during the wildfire events, and therefore, meteorological data had to be used as a proxy for exposure. In addition, other air quality data, information on the relative impact of differing allergy seasons, and differences in respiratory infection rates from the pre-fire through post-fire period were also not available. For example, North Carolina did not start collecting or monitoring pollen data until 2012. It is possible that differences between the pre-fire, during-fire, and post-fire periods had an impact on our data, but the only method available to us to control for confounding by these variables was to have our analyses restricted to the same calendar dates over the pre-fire through post-fire period in an effort to gain some control over these types of confounding factors. Additionally, the specific resident location was not available, and thus it was assumed that patients lived in close proximity to their local clinic. As a result, misclassification of exposures could have occurred and impacted this assessment. However, typically, exposure misclassification results in a bias towards the null, which would imply that the association of decreased respiratory performance with wind direction was likely stronger than what was detected in this study. In addition, this study limited its assessment to each fire event and did not assess effects longer than one-year post-fire or assess the cumulative effect of multiple fires. Due to these limitations, this study cannot assess causality but rather is an observational study that can provide some insight into possible important impacts of wildfires that support the worthiness of subsequent investigations using more controlled studies in the future.

## 5. Conclusions 

This study demonstrated an effect of reduced respiratory peak flow performance among patients at a local allergy clinic one year after suspected exposure to wildfire smoke. If confirmed in the future with more controlled studies, this finding has important clinical and policy implications, including the recognition of longer-term effects of smoke from wildfires. The impact of wildfire smoke on public health will continue to be a challenge as climate change contributes to the increase in frequency and magnitude of wildfires around the world.

## Figures and Tables

**Table 1:** Clinic patient demographics: Fire #1 had 527 total patients and Fire #2 had 315 total patients.

	Fire #1 Patient Demographics (%)	Fire #2 Patient Demographics (%)
**Age Distribution**		
<13 years old	30	40
13–17 years old	15	16
18–30 years old	8	6
31–49 years old	18	15
50–70 years old	21	17
>70 years old	8	6
**BMI (kg/m^2^)**		
<18.5 (underweight)	19	23
18.5–24.9 (normal)	28	30
25–29.9 (overweight)	26	23
30 or greater (obese)	27	24
**Sex**		
Male	44	49
Female	56	51
**Race**		
Black	26	30
White	73	66
Other	1	4
**Ethnicity**		
Hispanic	1	1
Non-Hispanic	99	99

County information where clinic is located: The county where the clinic was located had a population with roughly 15% over 65 years of age, a median age of 37.8 years old, the largest number of people were in the age range of 45–54 years old, 61% were white, 38% were African American, males and females were roughly equal percentages, the percent uninsured ranged from 10.9% to 29.5% depending on the specific census tract, percent in poverty ranged from 8% to 44% by census tract, unemployment ranged from 2% to 16% by census tract, and the overall median annual income for the county was $45,750.

**Table 2:** Average clinic measured difference (delta_PF) in peak flow (L/min) from predicted peak flow for Fire #1 across all time periods (pre-fire, during fire, post-fire) for specific groups of patients with allergic rhinitis and/or asthma. See Figure 1 for dates of each period.

	Pre-Fire Period	During-Fire Period	Post-Fire Period
	Average Delta_PF (*n*)	Average Delta_PF (*n*)	Average Delta_PF (*n*)
All patients	−44.67 (231)	−35.19 (498)	−60.43 (223)
Women only	−55.68 (132)	−53.45 (286)	−75.65 (125)
Men only	−29.97 (99)	−10.55 (212)	−41.03 (98)
Black only	−57.53 (52)	−37.5 (126)	−73.36 (55)
White only	−40.82 (177)	−36.34 (362)	−57.46 (162)

**Table 3:** Repeated measures ANOVA with Tukey pairwise comparisons of the delta_PF value (clinic measured vs. predicted) for patients with peak flow measures recorded during the pre-fire, during-fire, and post-fire time periods. The analysis was performed separately for each fire.

	Least Squares Mean Delta_PF (Liters per Minute)	*p*-Value for Comparisons of Delta_PF by Time Periods
Fire #1 (*n* = 378)		
Pre-fire	−35.00	
During fire	−23.84	
Post-Fire	−63.79	
Pre-fire vs. During fire		0.11
Pre-fire vs. Post-fire		<0.001
During fire vs. Post-fire		<0.001
Fire #2 (*n* = 162)		
Pre-fire	−45.42	
During fire	−37.00	
Post-Fire	−55.33	
Pre-fire vs. During fire		0.40
Pre-fire vs. Post-fire		0.32
During fire vs. Post-fire		0.07

**Table 4:** Adjusted multivariate linear regression with an outcome variable of delta_best during the post-fire period (i.e., clinic measured peak flow in liters per minute minus the patient’s personal best peak flow), with predictors including meteorological parameters of the percentage of time the wind was from the northwesterly direction during fire blowing from fire towards the town (light wind was a Beaufort scale of 3 = 1.5–5.5 m/s; Beaufort 4–5 = 5.5–10.7 m/s = moderate wind; Beaufort > 6 = >10.7 m/s = strong wind) (note: northwesterly wind is the direction the wind was coming from, meaning the wind was blowing from the northwest to the southeast. In other words, from the fire to the community downwind).

	Fire #1 Delta_Best (*n* = 264)		Fire #2 Delta_Best (*n* = 120)	
Meteorological Metric 3-Day Period before Clinic Visit When Town was Downwind of Fire	*p*-Value	Parameter Estimate (β)	*p*-Value	Parameter Estimate (β)
Any wind	0.032 *	−2.21	0.46	−0.58
No_wind	0.77	0.097	0.37	0.46
Light_wind	0.0010 *	−7.13	0.58	3.05
Moderate_wind	0.52	−0.87	0.64	−0.50
Strong_wind	0.0060 *	−13.90	0.10	−4.33

* Statistically significant. Adjusted for age, BMI, sex, and race.

## Data Availability

The datasets used and/or analyzed during the current study are available from the corresponding author upon reasonable request pending IRB approval.

## Abbreviations

BMI = Body Mass Index; ICD and ICD-9 = International Classification of Disease Codes version 9; IRB = Institutional Review Board; NOAA = National Oceanographic and Atmospheric Administration; SAS = Statistical Analysis System (software); ANOVA = analysis of variance; Delta_PF = the actual peak flow measured at the clinic visit minus the peak flow that was expected; Delta_Best = A derived variable referred to as “delta_Best” was the actual peak flow measured during the clinic visit minus their personal baseline best peak flow value.
